# Supplementary material for: Characterisation of Bioactive Peptides from Red Alga Gracilariopsis chorda
Source: Mar Drugs. 2023 Jan 11;21(1):49. doi: 10.3390/md21010049 (PMC9864793; doi:10.3390/md21010049)
Supplement: Supplementary file 1 [file marinedrugs-21-00049-s001.zip › marinedrugs-2148440-supplementary.pdf]

Supplemental data

(a)

PE-α

MKSVITTVISAADAAGRFPTSSDLESVQGNIQRAAARLEAAEKLADNHDAVVKEAGDACFAKYSYLKNSGEAGENQEKINKCYRDIDHYMRLINYS
LVVGGTGPLDEWGIAGAREVYRALNLPTASYVAAFAFTRDRLCVPRDMSAQAGVEYNSALDYVINSLS

PE-β

MLDAFSRVVVNSDAKAAYVGGSDLQALKTFIAEGNKRLDAVNSIVSNASCIVTDAVSGMICENPGLISPGGNCYTNRRMAACLRDGEIILRYVSYA
LLAGDPSVLEDRCLNGLKETYIALGVPTNSSVRAVSIMKAAAVAFISNTASQRKMDTTSGDCSALSSEIASYCDRVCSAIS

PE-γ

MDSPAFAVNGMFSAVNVGTSSFTKNNVTSQRTTASPAAVRMAVDPFQKQFQSPGKINVDYSRPKKLATYKRSGYSAILDYPTQTSMAGHYSISNCN
ITSGTKKILMKYDEYCAKGMMQVYKRSAVPYGEYTTKCTEGTLPQQAFDKRVFNRTQAFRQAQKPINVRLGEQYENRRLAFIFTNGCHREEQQFKE
MPMSTATYLAGRSEALGTCYRLVTPTTVAEDYMADSVRSQITQKAHPTGVYRVGVCEDGYAKGDAENRRVAALASEFRTSQQSASAITGQQYESAR
TARKLYASSCHHEETQIYQYPAVAAAMCRD

(b)

PC-α

MSIITKSIVNADAEARYLSPGELDRIKSFVLSGQRRLRIAQILTDNRELIVKQGGQQLFQKRPDVVSPGGNAYGEEMTATCLRDLDYYLRLVTYGI
VAGDVTPIEEIGLVGVKEMYNSLGTPISGVAEGVRSMKTVACSLLSGEDSAEAGFYFDYTLGAMQ

PC-β

MQDAITSVINAADVQGKYLDDNSLDKLRGYFQTGELRVRASATIAANAATIIKDSVAKALLYSDITRPGGNMYTTRRYAACIRDLDYYLRYATYGM
LAGDPSILDERVLNGLKETYNSLGVPIGATIQAIQAMKEVTSNLVGPDAGKEMGVYFDYVCSGLS

(c)

APC-α

MSIITKSIVNADAEARYLSPGELDRIKSFVLSGQRRLRIAQILTDNRELIVKQGGQQLFQKRPDVVSPGGNAYGEEMTATCLRDLDYYLRLVTYGI
VAGDVTPIEEIGLVGVKEMYNSLGTPISGVAEGVRSMKTVACSLLSGEDSAEAGFYFDYTLGAMQ

APC-β

MQDAITSVINAADVQGKYLDDNSLDKLRGYFQTGELRVRASATIAANAATIIKDSVAKALLYSDITRPGGNMYTTRRYAACIRDLDYYLRYATYGM
LAGDPSILDERVLNGLKETYNSLGVPIGATIQAIQAMKEVTSNLVGPDAGKEMGVYFDYVCSGLS

APC-γ

MSLISQIIVNADNELRYPSIGELQSINSYLKTGEKRINISCILRDKEKEIIQQASKTIFQIHPEYIAPGGNAEGARKRSLCLRDYSWYLRLITYGI
LAGDKNSIEKIGIIGVREMYNSLGVPIIGMIDAIDCLKKATAEFLNEEEIIIVAPYFDFIIQGMS

(d)

RuBisCo-L

MSQSVEERTRIKNERYESGVIPYAKMGYWDPDYVVKDTDVLALFRVSPQPGVDPVEASAAVAGESSTATWTVVWTDLLTACDLYRAKAYKVEAVPN
TTDQYFAFIAYDIDLFEEGSIANLTASIIGNVFGFKAVKALRLEDMRIPVAYLKTFQGPATGLVVERERMDKFGRPFLGATVKPKLGLSGKNYGRV
VYEGLKGGLDFLKDDENINSQPFMRWKERFLYSMEGVNRAIAASGEVKGHYMNITAATMEDMYERAEFAKQLGTVIIMIDLVIGYTAIQTMAIWAR
KNDMILHLHRAGNSTYSRQKIHGMNFRVICKWMRMAGVDHIHAGTVVGKLEGDPLMIRGFYNTLLQTHLKVNLPQGIFFEQDWASLRKVTPVASGG
IHCGQMHQLLDYLGNDVVLQFGGGTIGHPDGIQAGATANRVALESMVMARNEGRDYVAEGPEILRVAAKTCGPLQTALDLWKDITFNYTSTDTADF
VETPTANV

RuBisCo-S

MFEINSCRKQHSNVYIKVNAFDNTRGTESCVLSFIINRPAYEPGFELVRTEDDSRNQRYSFRSYATAKPEGSRY

Figure S1a-d: Amino acid sequences of the main WSP of G. chorda, namely PE-α, -β, and -γ (a), PC-α and -β (b), APC-α, -β, and -γ (c), and RuBisCo-L and -S (d).

Table S1. Amino acid composition and distribution of *G. chorda* phycobiliproteins and RuBisCo.

Amino acid	PE			PC		APC			RuBisCo	
	α	β	γ	α	β	α	β	γ	L	S
Ala	15.3	15.3	11.3	13	15.1	7.5	11.8	6.2	9.4	5.4
Val	5.5	8.5	6	1.9	8.2	8.1	6.2	2.5	8.2	5.4
Met	1.8	2.8	3.4	2.5	3.5	3.1	3.1	2.5	3.9	1.3
Ile	6.1	5.6	3.1	6.8	5.8	6.8	6.8	17.4	5.7	5.4
Leu	8.5	8.5	3.5	8.6	9.9	9.9	9.3	8.1	7.8	2.7
Phe	2.4	1.7	3.8	2.5	2.3	2.5	1.2	2.5	4.1	6.7
Pro	1.8	1.7	4.1	3.7	1.7	3.1	2.5	3.1	3.7	4.1
Try	0.6	0.0	0.0	0.6	0.0	0.0	0.0	0.6	1.6	0.0
Total hydrophobic amino acids	42.0	44.1	35.2	39.6	46.5	41.0	40.9	42.9	44.4	31.0
Arg	6.1	5.6	6.9	4.3	5.8	6.2	5.6	5	5.3	10.8
Glu	6.1	2.8	5	5.6	4.1	6.8	3.1	8.1	5.5	8.1
Gln + Asn	6.7	7.3	10.7	9.8	11.1	6.8	7.4	8.7	7.2	10.8
Lys	4.3	2.8	5.6	3.7	2.9	3.7	4.4	6.2	5.1	4.1
His	1.2	0.0	1.6	0.6	0.0	0.0	0.0	0.6	2.0	1.4
Asp	6.7	6.2	3.5	2.5	5.8	5.6	8.1	4.3	6.0	4.1
Total hydrophilic amino acids	31.1	24.7	33.3	26.5	29.7	29.1	28.6	32.9	31.1	39.3
Ser	7.9	12.4	8.2	9.9	7.6	7.5	6.2	7.4	3.9	10.8
Gly	7.9	7.4	5.7	8.0	6.4	10.6	8.7	7.4	8.8	4.0
Thr	3.7	4.0	8.8	8.0	5.2	5.6	6.8	2.5	6.8	5.4
Tyr	5.5	3.4	6.3	6.8	2.9	5.0	7.5	5.0	4.1	6.8
Cys	1.9	4.0	2.5	1.2	1.7	1.2	1.3	1.9	0.9	2.7
Total neutral amino acids	26.9	31.2	31.5	33.9	23.8	29.9	30.5	24.2	24.5	29.7
Total aromatic amino acids	8.5	5.1	10.1	9.9	5.2	7.5	8.7	8.1	9.8	13.5
pI	5.19	5.38	9.32	6.57	5.13	4.9	5.1	5.51	6.11	8.68
MW in Da	17722.9	18333.8	35467.9	17529.7	18294.9	17522.0	17470.8	18133.1	54194.1	8662.6
